# A novel optimization approach incorporating non-stomatal limitations predicts stomatal behaviour in species from six plant functional types

**DOI:** 10.1093/jxb/erz020

**Published:** 2019-02-04

**Authors:** Teresa E Gimeno, Noelia Saavedra, Jérôme Ogée, Belinda E Medlyn, Lisa Wingate

**Affiliations:** 1 INRA, UMR ISPA, Villenave d’Ornon, France; 2 Basque Centre for Climate Change (BC3), Leioa, Spain; 3 IKERBASQUE, Basque Foundation for Science, Bilbao, Spain; 4 Department of Forest Ecology and Management, Swedish University of Agricultural Sciences, Umeå, Sweden; 5 Hawkesbury Institute for the Environment, Western Sydney University, Penrith, NSW, Australia

**Keywords:** Drought, fern, mesophyll conductance, ontogeny, optimization, photosynthesis, plant functional type, stomatal conductance, transpiration, water use efficiency

## Abstract

The primary function of stomata is to minimize plant water loss while maintaining CO_2_ assimilation. Stomatal water loss incurs an indirect cost to photosynthesis in the form of non-stomatal limitations (NSL) via reduced carboxylation capacity (CAP) and/or mesophyll conductance (MES). Two optimal formulations for stomatal conductance (*g*_s_) arise from the assumption of each type of NSL. In reality, both NSL could coexist, but one may prevail for a given leaf ontogenetic stage or plant functional type, depending on leaf morphology. We tested the suitability of two *g*_s_ formulations (CAP versus MES) on species from six plant functional types (C_4_ crop, C_3_ grass, fern, conifer, evergreen, and deciduous angiosperm trees). MES and CAP parameters (the latter proportional to the marginal water cost to carbon gain) decreased with water availability only in deciduous angiosperm trees, while there were no clear differences between leaf ontogenetic stages. Both CAP and MES formulations fit our data in most cases, particularly under low water availability. For ferns, stomata appeared to operate optimally only when subjected to water stress. Overall, the CAP formulation provided a better fit across all species, suggesting that sub-daily stomatal responses minimize NSL by reducing carboxylation capacity predominantly, regardless of leaf morphology and ontogenetic stage.

## Teaser

A model formulation that reduced carboxylation capacity to optimize water costs for carbon gain successfully predicted stomatal behaviour for species from six plant functional types, irrespective of water availability or leaf ontogeny.

## Introduction

Climate change is predicted to decrease water availability and increase drought risk in many regions around the world (Sheffield and Wood, 2007; Dai and Zhao, 2017). Water availability is one of the main factors regulating vegetation carbon and water fluxes and drought impacts on the vegetation, reducing productivity, changing species distribution, or even driving large-scale mortality events (Vicente-Serrano *et al.*, 2013; Lobell *et al.*, 2014; Allen *et al.*, 2015). The impact of drought on vegetation functioning is not well represented in current dynamic global vegetation models (DGVM), which still vary greatly in their underlying assumptions (Egea *et al.*, 2011; De Kauwe *et al.*, 2014; Rogers *et al.*, 2017). This is partly caused by the diversity of strategies deployed by plants to cope with drought, ranging from avoidance or migration to drought tolerance (Chaves *et al.*, 2002; Jump and Peñuelas, 2005). Drought tolerance or avoidance requires the coordination of morphological and physiological traits (Croker *et al.*, 1998; Brodribb and Holbrook, 2003; Bartlett *et al.*, 2016), and one of the most important and the most immediate physiological mechanism for regulating plant water loss is stomatal closure (Flexas *et al.*, 2006; Rodriguez-Dominguez *et al.*, 2016; Martinez-Vilalta and Garcia-Forner, 2017). Thus, understanding stomatal regulation is fundamental to projecting the impact of increased drought risk on the climate–vegetation system.

According to optimization theory, stomata should operate to maximize photosynthetic uptake minus the cost of water loss to the plant (Cowan and Farquhar, 1977). The direct benefit of stomatal opening consists of increasing the CO_2_ concentration at the sites of carboxylation, but the nature of the associated water costs remains unclear. Transpiration losses through the stomata lead to decreased leaf water potential (Ψ_leaf_), which triggers a cascade of processes affecting leaf conductivity, cell turgidity, protein stability, metabolic rates, solute accumulation, and membrane and cell wall permeability (Chaves *et al.*, 2002; Brodribb and Holbrook, 2003; Flexas *et al.*, 2012). As a consequence, non-stomatal limitations (NSL) to photosynthesis arise in the form of reduced carboxylation capacity or CO_2_ diffusivity through the mesophyll. As NSL arise, stomata should continue to operate optimally, but the sensitivities of stomatal and non-stomatal processes could be uncoupled, challenging our ability to predict stomatal behaviour. For example, changes in the capacitance and resistance pathway from the soil to the leaves can alter stomatal sensitivity to vapour pressure deficit (*D*_w_) (Martins *et al.*, 2016). Based on the idea that the cost of stomatal opening arises from NSL, Dewar *et al.* (2018) proposed two analytical formulations for stomatal conductance to CO_2_ (*g*_sc_). In these formulations, photosynthesis is maximized instantaneously and the cost of water use arises from reductions in photosynthesis resulting from decreasing Ψ_leaf_. These formulations can be structurally similar to previous ones (e.g. Katul *et al.*, 2010; Medlyn *et al.*, 2011; Prentice *et al.*, 2014), but their advantage is that they do not require a defined temporal framework over which stomatal behaviour is optimized. In addition, Dewar *et al.* (2018) provide a formulation for the elusive λ parameter (marginal water cost to carbon gain; Cowan and Farquhar, 1977) and testable predictions for this cost parameter as a function of hydraulic and photosynthetic traits. Dewar *et al.* (2018) hypothesized that NSL were induced by a reduction in either carboxylation capacity or mesophyll conductance (*g*_m_). In reality, such a dichotomy is probably rare and both types of NSL coexist (Zhou *et al.*, 2013; Drake *et al.*, 2017). Nevertheless, we could expect a certain NSL to prevail depending on the sensitivity of *g*_m_ and the photosynthetic machinery to Ψ_leaf_.

Leaves that are likely to experience drought during their lifespan exhibit morphological traits that contribute to maintain Ψ_leaf_ and physiological activity under water stress (Mediavilla and Escudero, 2003; Flexas *et al.*, 2006); one such trait is high leaf mass per area (LMA). High-LMA leaves often have multiple layers of cells with thick walls and tortuous mesophyll interspaces (Niinemets *et al.*, 2009; Onoda *et al.*, 2017). These traits protect the photosynthetic machinery from dehydration (Gimeno *et al.*, 2010; Limousin *et al.*, 2013) but they are also associated with low mesophyll diffusivity (Niinemets *et al.*, 2009). *g*_m_ can be the most important limitation to photosynthesis in evergreen trees and shrubs (Flexas *et al.*, 2012; Peguero-Pina *et al.*, 2016) and also in many ferns (Carriqui *et al*., 2015; Tosens *et al.*, 2016). Ferns have more rudimentary stomata, in the sense that they are insensitive to abscisic acid, CO_2_, and blue light (Brodribb and McAdam, 2011) and they appear to respond only to changes in vapour pressure deficit (Martins *et al.*, 2016). Franks and Britton-Harper (2016) observed that stomata of some ferns can close in response to elevated CO_2_, but these responses have not been tested under the framework of optimization theory.

In addition to drought tolerance and leaf morphology, longevity and construction costs can also influence stomatal behaviour. Lin *et al.* (2015) evaluated the convergence across plant functional types (PFTs) in their ability to operate stomata optimally, and found that PFTs with low marginal water use per unit of C gain had water-transport systems with greater construction costs, except for species with a C_4_ pathway, for which optimization theory predicts the lowest marginal water cost. In addition to an associated cost to water transport, leaf construction costs could also influence stomatal regulation. Leaf construction costs depend on leaf lifespan and turnover: generally, the longer the lifespan, the slower the return of nutrient and dry mass investment (Wright *et al.*, 2004). Leaf development is a crucial stage for all PFTs and in deciduous species, it can occur over a significant fraction of their lifespan. Yet, only mature fully expanded leaves are targeted for the vast majority of physiological measurements (with some exceptions, e.g. Field and Mooney, 1983; Medlyn *et al.*, 2002; Locke and Ort, 2014; Macinnis-Ng *et al.*, 2017), and hence DGVM assume constant stomatal behaviour throughout leaf ontogeny (Keenan *et al.*, 2013). During leaf construction, respiratory costs are high, so that net photosynthesis per unit of water transpired is usually low (Rajaona *et al.*, 2013; Jensen *et al.*, 2015). Additionally, during development, the internal leaf anatomy experiences modifications including cell multiplication and expansion, increase in the number of chloroplasts, thickening of the cell wall, and formation of the intercellular air spaces (Niinemets *et al.*, 2009; Kuusk *et al*., 2018). Collectively, these modifications should result in greater carboxylation capacity and the ability to maintain greater Ψ_leaf_ under drought stress, but also lower *g*_m_ (Aasamaa *et al.*, 2005; Grassi and Magnani, 2005; Barbour *et al.*, 2016). Hence, in developing leaves the photosynthetic costs of stomatal opening likely result from a decrease in carboxylation capacity, rather than in *g*_m_.

Here, we tested the formulations proposed by Dewar *et al.* (2018) to determine when carboxylation capacity (the CAP formulation) or *g*_m_ (the MES formulation) are the predominant NSL to photosynthesis. We expected developing leaves to have a greater marginal water cost per unit of C gain than mature leaves. We also hypothesized that in developing leaves the carboxylation capacity should be more sensitive to Ψ_leaf_ and the CAP formulation should fit better. Regarding the effect of leaf morphological differences among species from various PFTs, we hypothesized that in leaves with lower LMA the photosynthetic machinery would be more sensitive to fluctuations in Ψ_leaf_ and the CAP formulation would be more suitable, while in species where *g*_m_ constitutes the main limitation to photosynthesis, such as in evergreen species with greater LMA and ferns, the MES formulation could fit better. On the other hand, in leaves with low maximum realized *g*_m_, fluctuations in *g*_m_ would have a marginal effect and NSL should arise from limited carboxylation capacity. We address these hypotheses within the theoretical framework of Dewar *et al.* (2018) by comparing stomatal behaviour between mature and developing leaves of species from six PFTs (including a fern) maintained under two watering regimes.

## Materials and methods

### Plant material and experimental design

We selected seven species economically or ecologically relevant to the ecosystems in the south-west of France and representative of six PFTs: a fern, common bracken (*Pteridium aquilinum* L. Kuhn); an evergreen conifer, maritime pine (*Pinus pinaster* Ait.); two deciduous angiosperm temperate trees, pedunculate oak (*Quercus robur* L.) and silver birch (*Betula pendula* Roth); a C_3_ grass, purple moor grass [*Molinia caerulea* (L.) Moench]; an evergreen angiosperm tree, cider gum (*Eucalyptus gunnii* Hook.f.); and a C_4_ crop, maize (*Zea mays* L.).

Saplings of *P. pinaster*, *Q. robur*, *B. pendula*, and *E. gunnii* were grown from seeds obtained from nearby plantations in an open-air nursery at INRA Pierroton (Cestas, France; annual precipitation 977 mm, annual mean temperature 13 °C). Plants of *M. caerulea* and *P. aquilinum* were grown from tussocks (~15 cm diameter) and overwintering rhizomes, respectively, collected in February 2015 from a local forest (Le Bray experimental site; Wingate *et al.*, 2010). Plants of *Z. mays* (variety DKC 5784) were grown from seeds sown in May 2015. All species were grown in 3.4 l square pots. The soil substrate consisted of a 4:2:1 (v/v) mix of bark:peat:soil (typical sandy soil from the Le Bray experimental site). A slow-release fertilizer (Osmocote^TM^, Mashville, OH, USA) was added at the beginning of the experiment.

From March (May for *Z. mays*) to September 2015, pots were kept in a glasshouse at the INRA campus of La Grande Ferrade (Villenave d’Ornon, France). Pots were watered every other day to field capacity with an automatic dripping system. In March and April 2015, to treat a fungal infection by *Erysiphe* sp., *Q. robur* plants were sprayed twice with a 0.4 g l^−1^ solution of tebuconazole and twice with a 0.6 g l^−1^ solution of Meptyldinocap Karathane® 3D (Merck KGaA, Darmstadt, Germany). In addition, in May 2015, an early aphid outbreak on *B. pendula* was controlled by spraying plants with colza oil. Climatic conditions were monitored inside the glasshouse with a temperature and humidity probe (HMP60, Vaisala, Vanta, Finland) and, in June and July, a quantum sensor (SQ-200, Apogee, Logan, UT, USA). Ten-minute averages were logged on a 21X micrologger® (Campbell Scientific, Logan, UT, USA). Mean temperature over the study period inside the glasshouse was 21.7 °C during the day and 16.6 °C at night. A shading cloth was permanently deployed from May 2015 and mean daily photosynthetic photon flux density was 121±6.4 mol m^−2^ d^−1^ (measured over 38 days in June–July).

In July 2015, we selected seven plants of each species and assigned them to a low-water-availability treatment. The concept of low water availability is complex, and attaining a homogeneous level of water stress that allows for comparison between species is not straightforward (Drake *et al.*, 2017). Our low-water treatment consisted of cessation of watering for a number of days until the mean predawn leaf water potential (Ψ_pd_) was reduced by half of the total range. We determined this range and number of days for our study species in a separate experiment. In brief, five randomly selected individuals of each species were placed in a climatically controlled chamber (MD1400, Snijders Labs, Tilburg, The Netherlands) on 1 July 2015. The temperature and relative humidity inside the chamber were set to mimic a typical summer day inside the glasshouse, with a 13/9 h light/dark cycle and a photosynthetic photon flux density of 580 µmol m^−2^ s^−1^, supplied by fluorescent lamps (BriteGro 2084, Sylvania, BioSystems, Wageningen, The Netherlands). Plants inside the chamber were watered to field capacity on the first day and then watering was withheld until all individuals died. We assessed dead individuals as plants that had no green leaves and had lost stem flexibility, and verified that none of these individuals re-sprouted following re-watering. Pots with *M. caerulea* tussocks were measured until at least 80% of the leaves in a pot had completely withered. After 25 days of withholding water, individuals of *P. pinaster* still showed no visible signs of water stress; by this point Ψ_pd_ had dropped from –0.32±0.04 to –1.19±0.11 MPa (Table 1) and the experiment ceased. The range of Ψ_pd_ for each species was determined as the difference between the mean (*n*=5 plants per species) maximum and minimum Ψ_pd_. Measurements of Ψ_pd_ were made with a Scholander-type pressure chamber (SARL SAM PRECIS 2000, Gradignan, France).

**Table 1.** Mean ±SE (n=5–7) predawn leaf water potential (Ψ_pd_) for the study species in the trial experiment and in the August 2015 campaign under the well-watered (WW) and low water availability (LW) regimes

Species	Trial experiment		August 2015	
	Max Ψ_pd_ (MPa)	Min Ψ_pd_ (MPa)	Ψ_pd_ WW (MPa)	Ψ_pd_ LW (MPa)
*B. pendula*	–0.56±0.1	–2.31±0.25	–0.51±0.12	–1.47±0.34
*E. gunnii*	–0.58±0.28	–2.03±0.3	–0.58±0.28	–0.97±0.4
*M. caerulea*	–0.46±0.04	–2.24±0.22	–0.48±0.05	–1.38±0.25
*P. pinaster*	–0.32±0.04	–1.19±0.11	–0.37±0.1	–0.54±0.08
*P. aquilinum*	–0.47±0.13	–1.24±0.06	–0.45±0.07	–0.89±0.25
*Q. robur*	–0.46±0.21	–2.44±0.2	–0.53±0.09	–0.83±0.24
*Z. mays*	–0.28±0.03	–1.94±0.26	–0.4±0.05	–1.42±0.29

In August 2015, there were no significant differences among species (*F*=1.9, *P*=0.11) and plants in the LW treatment had significantly lower Ψ_pd_ (*F*=15.9, *P*<0.01).

### Morphological and physiological measurements

LMA was calculated from one mature and one young developing leaf collected from five individuals per species in mid-June 2015. Mature fully expanded leaves were sampled from the middle of the plant (in height), and young developing leaves, not fully expanded, from the upper third. For *P. aquilinum*, we selected a pinna (leaflet) close to the base of the frond as a mature pinna, and the most distal as a young pinna. Sampled leaves were photographed and dried at 70 °C for 48 h. Leaf area was calculated using ImageJ software for image analysis (Rasband, 2009). Leaf thickness was measured on the same leaves.

**Fig. 1.**
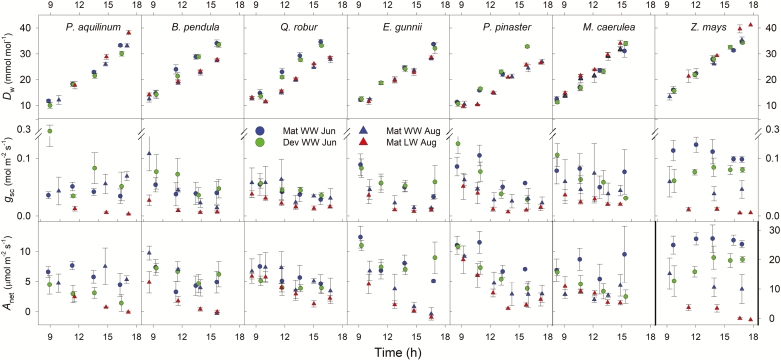
Mean ±SE (*n*=3–5) vapour pressure deficit (*D*_w_), stomatal conductance to CO_2_ (*g*_sc_), and photosynthesis (*A*_net_) measured in mature (Mat) and developing (Dev) leaves and in well-watered (WW) and low-watered (LW) plants during the day. Circles and triangles depict measurements from the June 2015 and August 2015 campaigns, respectively. Different shades correspond to measurements on WW versus LW plants or to measurements on mature versus developing leaves. Note the change in scale for *A*_net_ in the *Z. mays* panel.

We conducted two gas-exchange campaigns, each over the course of 3 or 4 sunny and cloudless days with comparable conditions within campaigns. In the first campaign (18, 22, and 24 June 2015), we measured five well-watered individuals of each species, and during the second campaign (5, 6, 20, and 21 August 2015), we measured three or four individuals of each species and water treatment (well-watered and low-watered). On each day, we measured gas exchange starting at 08.00 h (local time) every 2–2.5 h on the same set of plants, to track net photosynthesis (*A*_net_) and *g*_sc_ along a gradually increasing gradient of *D*_w_ (Fig. 1). Plants measured each day were kept outside in an open area next to the glasshouse, exposed to full sun, from the first until the last round of measurements. A mature fully expanded leaf, and in June also a young developing leaf, was measured on each individual at each measurement round. Gas-exchange measurements were performed with an open-flow portable photosynthesis system (IRGA, LI-6400, LI-COR, Lincoln, NE, USA) with a standard leaf chamber fluorometer head (LI-6400–40). We measured *A*_net_ and *g*_sc_ under saturating light intensity (1800 µmol m^−2^ s^−1^ for *Z. mays* and 1500 µmol m^−2^ s^−1^ for all other species) provided by the inbuilt LI-6400 red–blue LED lamp. The CO_2_ concentration entering the cuvette was set to 390 µmol mol^−1^ using the inbuilt CO_2_ control unit, and airflow through the cuvette was set to 500 µmol s^−1^ (300 µmol s^−1^ when *g*_sc_ <0.03 mol m^−2^ s^−1^). Relative humidity and temperature inside the leaf cuvette were maintained as close as possible to ambient conditions during each measurement period. Measurements were logged after reaching steady state (within 3–5 min).

In August, we measured predawn leaf (*B. pendula*, *Q. robur*, *M. caerulea*, *Z. mays*), needle (*P. pinaster*), branch (*E. gunnii*), or pinna (*P. aquilinum*) water potential (Ψ_pd_) on all water-stressed plants (*n*=7 per species) and on the well-watered plants selected for gas exchange measurements in this campaign (*n*=3 or 4 per species, except for well-watered *E. gunnii*, where Ψ_pd_ was assumed to be the maximal Ψ_pd_ from the trial experiment).

### Data analyses and model fitting

We tested the effects of leaf age, species, and water stress on optimal stomatal behaviour according to the formulations of Dewar *et al.* (2018). When reduced carboxylation capacity (CAP) underlies the costs of stomatal opening, Dewar *et al.* (2018) predict that *g*_sc_ (stomatal conductance to CO_2_ in mol m^−2^ s^−1^) should follow:

$$\begin{array}{l} g_{sc}=g_{0}+(1+\frac{\xi }{\sqrt{D_{w}}})\frac{A_{net}}{C_{a}-\Gamma^{*}} \end{array}$$ (1)

where *D*_w_ (mmol mol^−1^) is the leaf-to-air vapour pressure deficit, *A*_net_ (µmol m^−2^ s^−1^) is net photosynthesis, *C*_a_ (µmol mol^−1^) is the ambient CO_2_ concentration, Γ* (µmol mol^−1^) is the CO_2_ photorespiratory compensation point according to Bernacchi *et al.* (2001), and ξ (mmol^0.5^ mol^−0.5^) is a fitting parameter proportional to the square root of λ (Cowan and Farquhar, 1977) and equivalent to the *g*_1_ parameter in Medlyn *et al.* (2011). The intercept *g*_0_ (mol m^−2^ s^−1^) is another fitting parameter that was added to the model to represent suboptimal water losses through leaf cuticles or leaky stomata (Medlyn *et al.*, 2011). Parameters ξ and *g*_0_ were estimated from non-linear least-square regressions using the ‘nls’ function in R (R Development Core Team, 2017). The non-linear nature of the CAP formulation does not allow a straightforward comparison among species and between treatments. Therefore, to assess differences in ξ among species, between leaf ages (June campaign) and between watering treatments (August campaign), we compared the 95% confidence intervals (CI) for the fitted parameter (ξ; Gimeno *et al.*, 2016). Our estimates of ξ (in mmol^0.5^ mol^−0.5^ and calculated for *g*_sc_) can be related to the *g*_1_ estimates of Lin *et al.* (2015, in kPa^0.5^ and calculated for *g*_s_ to water) according to $g_{1}=\xi /1.6\sqrt{P}$, where *P* is atmospheric pressure and the factor 1.6 accounts for the ratio of the molecular diffusion coefficients for CO_2_ and water.

In addition, according to the CAP formulation, Dewar *et al.* (2018) also predict that parameter ξ should vary according to:

$$\begin{array}{l} \xi \sqrt{V_{cmax0}}=\sqrt{\frac{K_{sl}\left|\Psi_{c}\right|k_{m}+\Gamma^{*}}{1.6}} \end{array}$$ (2)

where *V*_c max0_ is the maximum carboxylation capacity in the absence of NSL, *k*_m_ is the Michaelis constant for CO_2_ (710 μmol mol^−1^; Bernacchi *et al.*, 2001), and Ψ_c_ is the critical leaf water potential when photosynthetic capacity is zero (–2 MPa; Dewar *et al.*, 2018). Noting that Dewar *et al.* (2018) neglect mitochondrial respiration, *V*_cmax_=0 at Ψ_leaf_=Ψ_c_ also translates into *A*_net_=0. We compared our estimates of ξ $\sqrt{{\hat{V}}_{cmax0}}$ with the predicted values according to equation 2 for a range of predawn Ψ (Ψ_pd_, from 0 to –2 MPa). To do so, we estimated temperature-corrected *V*_cmax0_ (${\hat{V}}_{cmax0}$) with the one-point method (De Kauwe *et al.*, 2016) from mid-morning gas-exchange measurements (see Supplementary Protocol S1 at *JXB* online). The soil-to-leaf hydraulic conductance (*K*_sl_) was estimated from equations 5b and 5c in Dewar *et al.* (2018) using a range of fixed values of the root-to-leaf hydraulic conductance (*K*_rl_=2, 5, 12, and 50 mmol m^−2^ s^−1^ MPa^−1^, Dewar *et al.,* 2018), and a soil-to-root conductance estimated from retention curve parameters typical of an organic soil (Ogée and Brunet, 2001) and a bulk density of 0.25 g cm^−3^.

When decreased mesophyll conductance (MES) underlies the cost of stomatal opening, Dewar *et al.* (2018) predict:

$$\begin{array}{l} g_{sc}=g_{0}+\sqrt{\frac{E_{max}A_{net}}{1.6D_{w}(C_{a}-\Gamma^{*})}} \end{array}$$ (3)

where

$$\begin{array}{l} E_{max}=K_{sl}(\Psi -\Psi_{c}) \end{array}$$ (4)

and Ψ_c_ represents here the critical leaf water potential that leads also to *A*_net_=0 but through a reduction of *g*_m_ rather than *V*_cmax_. We estimated *E*_max_ from the square of the slope of the linear relationship between *g*_sc_ and $\sqrt{\frac{A_{net}}{1.6D_{w}(C_{a}-\Gamma^{*})}}$ (hereafter the MES index) for each species, leaf age, and watering treatment. We used analyses of homogeneity of slopes to test for the effects of species, leaf age (June campaign), and water stress (August campaign) on $\sqrt{E_{\max}}$. Additionally, we compared our estimates of *E*_max_ with the predicted value according to equation 4 for the same range of Ψ_pd_ (0 to –2 MPa and assuming Ψ_s_=Ψ_pd_) and *K*_sl_, calculated from four *K*_rl_ (2, 5, 12, and 50 mmol m^−2^ s^−1^ MPa^−1^; Dewar *et al.*, 2018) as explained above.

We fitted equations 1 and 3 to our gas-exchange data and used the likelihood ratio test, the Akaike Information Criterion (AIC, with ΔAIC>10 indicating an improvement in the model fit), and root mean square error (RMSE) from the relationship between observed and predicted *g*_sc_ to compare between formulations for each species, leaf age, and watering treatment. We assessed differences among species and between leaf ages on morphological parameters (LMA and leaf thickness, June campaign) and among species and between watering treatments on Ψ_pd_ (August campaign) with two-way ANOVA. We tested for the overall effect of species and leaf age (June campaign) or watering treatment (August campaign) on *A*_net_ and *g*_sc_ with linear mixed models including plant and round of measurements as random factors (Zuur *et al.*, 2009). We tested for the effect of leaf age (June campaign) and watering (August campaign) on ξ and *E*_max_ with one-way ANOVA. Finally, we explored the relationship between ξ and *E*_max_ and LMA and leaf thickness, for different species and leaf ages (only the June campaign), using linear regression. All analyses were performed in R v3.4.3 (R Development Core Team, 2017).

## Results

The seven study species exhibited morphological trait values representative of their corresponding PFT (*F*=60.1 for LMA and 38.6 for thickness, *P*<0.001; Supplementary Fig. S4). In all species, LMA and leaf thickness were significantly lower in developing leaves than mature leaves (*F*=11.1, *P*=0.002 for LMA and *F*=16.2, *P*<0.001 for leaf thickness; Supplementary Fig. S4).

Over the course of the gas-exchange campaigns, *D*_w_ increased gradually from 8 to 42 mmol mol^−1^. For all species and in both campaigns, we measured *A*_net_ and *g*_sc_ under a *D*_w_ range of at least 12–35 mmol mol^−1^ (Fig. 1). We measured maximum *A*_net_ and *g*_sc_ on the first round of measurements, when *D*_w_ was minimal. In general, as *D*_w_ increased, *A*_net_, *g*_sc_, and estimated carboxylation capacity (${\hat{V}}_{cmax}$) decreased from mid-morning to midday measurements (Supplementary Fig. S1). The ratio of intercellular to ambient CO_2_ concentrations (*C*_i_/*C*_a_) was between 0.6 and 0.8 for the C_3_ species and remained relatively constant within species along an increasing *D*_w_ gradient in the June campaign (Supplementary Fig. S2). We measured the lowest *C*_i_/*C*_a_ in the C_4_ crop (*Z. mays*) under well-watered conditions (Supplementary Fig. S2). In August, under low water availability, *C*_i_/*C*_a_ gradually increased with *D*_w_ in *P. pinaster* and *Z. mays* (Supplementary Fig. S2). There were significant differences among species (*P*<0.05, except for *g*_sc_ in June, *P*=0.07) in *g*_sc_ and *A*_net_: the C_4_ crop (*Z. mays*) had significantly higher *A*_net_ and lower *g*_sc_ than the other species (Fig. 1). In June, developing leaves had significantly higher *g*_sc_ and lower *A*_net_ than mature fully expanded leaves, although the leaf age effect was species specific, with maximum differences in the C_4_ species for *A*_net_ and in the fern for *g*_sc_ (Fig. 1). In addition, developing leaves had lower ${\hat{V}}_{cmax0}$ (Supplementary Fig. S1). The low-water treatment reduced Ψ_leaf_, *g*_sc_, *A*_net_, and ${\hat{V}}_{cmax0}$ (Table 1, Fig. 1, Supplementary Fig. S1). Again, the treatment effect was species specific: the reduction in both *g*_sc_ and *A*_net_ under low-water treatment was least pronounced in the two evergreen trees (Fig. 1), to the point that Ψ_leaf_ of *P. pinaster* was only marginally affected (Table 1).

**Fig. 2.**
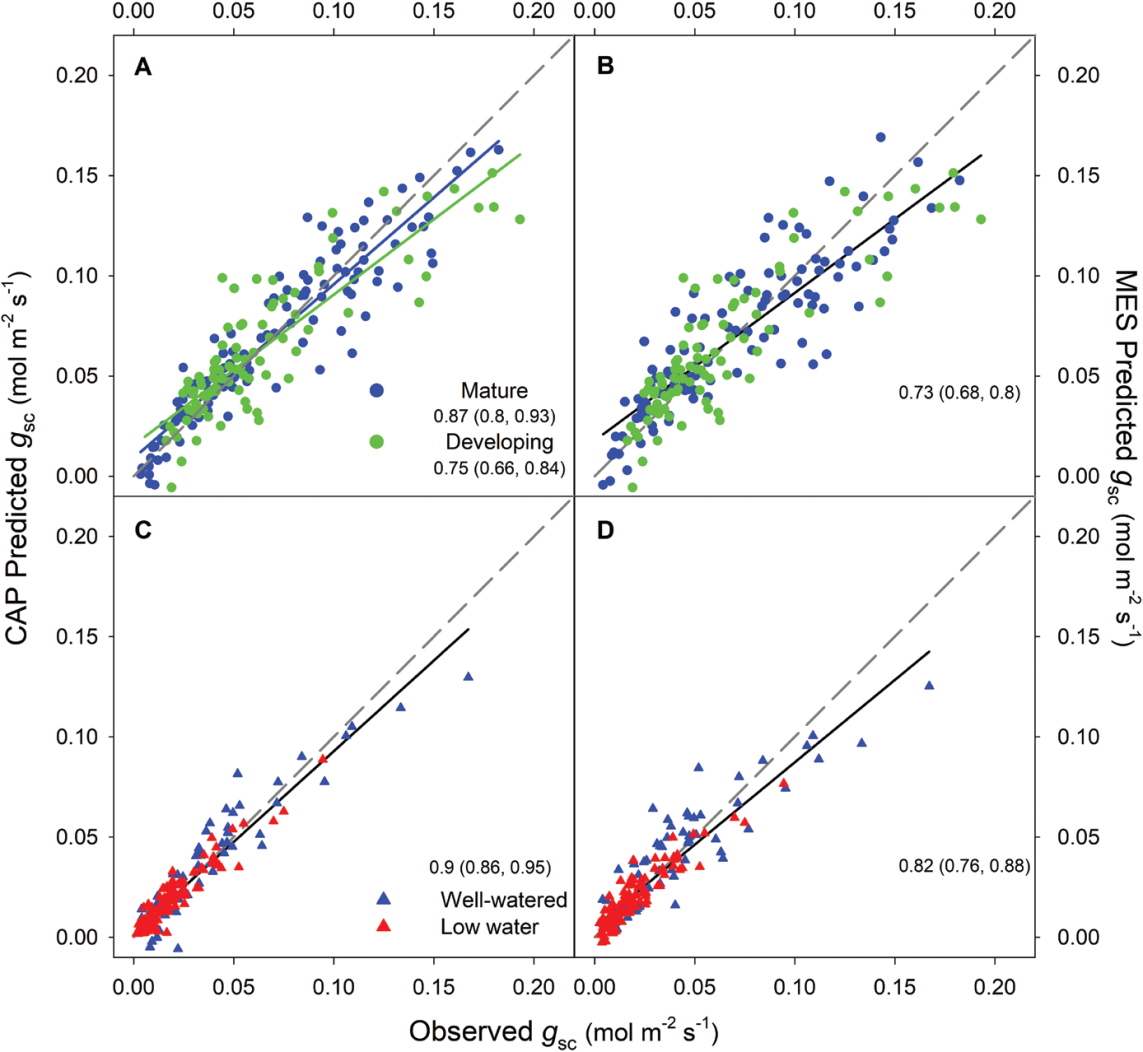
Relationship between predicted and observed stomatal conductance to CO_2_ (*g*_sc_) according to the CAP (A and C) and MES (B and D) formulations for mature and developing leaves (A and B) and under well-watered and low-watered conditions (C and D) for the study species for which the CAP and MES fits were significant. The dashed grey line depicts the 1:1 line. The slope of each linear relationship is indicated on each plot (with its corresponding 95% CI).

We found that both the CAP and the MES formulations fitted our data for most species, leaf ages, and watering treatments, with a few exceptions. Neither the CAP nor the MES formulation fitted the fern (*P. aquilinum*) data for either mature or developing leaves in the June campaign, or for well-watered ferns in the August campaign (Supplementary Fig. S3). Similarly, neither the CAP nor the MES formulation fitted the measurements on well-watered plants of *M. caerulea* (the C_3_ grass) in the August campaign (Supplementary Fig. S3). In addition, the CAP formulation did not fit the measurements on the C_4_ species *Z. mays* for developing leaves in June and under either watering treatment in August (Supplementary Fig. S3). For the other species, leaf ages, and watering treatments, both the CAP and MES formulations fitted our observations. When both the CAP and MES formulations could explain our data, the AIC value and the RMSE, as well as the likelihood ratio test (in most cases), indicated that the CAP formulation provided a better fit than that of the MES formulation, with the exception of developing leaves in *P. pinaster* and *Q. robur* (Table 2). Observed and predicted *g*_sc_ values were more closely related for the CAP formulation (lower RMSE), regardless of leaf age and watering regime (Fig. 2). For the CAP formulation, there was a significant difference between mature and developing leaves, with a smaller RMSE for mature leaves compared with developing leaves (Table 2). There were no significant differences in the fit between low-watered and well-watered plants (Fig. 2).

**Table 2.** Estimated intercept (g_0_) and slope parameters (±SE), RMSE, and AIC for the two formulations for stomatal conductance, CAP (ξ and g_1_) and MES (E_max_), for all the study species in mature and developing leaves (June 2015) and for well-watered and low watered plants (August 2015), and results of the model intercomparison

Species	Campaign	Leaf age	Treatment	CAP					MES				χ^2^
				*g*_0_ (mmol m^−2^ s )	ξ (mmol^0.5^ mol^−0.5^)	*g*_1_ (kPa^0.5^)	RMSE (%)	AIC	*g*_0_ (mmol m^−2^ s )	*E*_max_ (mmol m^−2^ s^−1^)	RMSE (%)	AIC	
*Z. mays*	June 2015	Mature	Well-watered	1.3±11.4	2±0.7	0.4±0.1	1.57	–120	9.4±18.5	**5.1±1.9**	1.89	–111	**8.44**
*B. pendula*				4.1±7.7	**7.7±1.9**	**1.5±0.4**	1.94	–90	–4.3±14.4	**6.4±3.2**	2.30	–72	<0.01
*Q. robur*				1.5±4.7	**6.2±1**	**1.2±0.2**	1.21	–108	–17.2±10.4	**7.7±2.4**	1.52	–86	<0.01
*E. gunnii*				3.2±7.8	**5.7±1.1**	**1.1±0.2**	1.76	–99	–19.5±13.7	**7.9±2.5**	2.11	–92	**7.31**
*M. caerulea*				–0.1±7.4	**9.8±0.9**	**2±0.2**	1.11	–92	**–31.4±12.9**	**15±3.5**	1.83	–77	**15.91**
*P. pinaster*				3.1±10.2	**6.9±1.3**	**1.4±0.3**	1.62	–81	–20.1±20.6	**9.3±3.7**	2.23	–70	**10.39**
*P. aquilinum*				**17.3±6.9**	1.3±1.5	0.3±0.3	0.99	–103	21.7±12.6	0.6±0.7	1.30	–93	**9.26**
*Z. mays*	June 2015	Developing	Well-watered	**16±4.8**	0.9±0.4	0.2±0.1	0.95	–143	–4.7±11.1	**5.4±1.4**	1.40	–125	**17.5**
*B. pendula*				–16.8±13.4	**14.8±2.9**	**2.9±0.6**	2.64	–74	–32.2 ± 21	**17±7.2**	3.16	–67	**6.45**
*Q. robur*				**21±5.1**	**4.7±1.7**	**0.9±0.4**	0.96	–123	**14.1 ± 6.6**	**2.8±1.2**	0.90	–126	**2.79**
*E. gunnii*				8.4±12	**5.3±1.9**	**1.1±0.4**	1.97	–84	–0.7±19.1	**5±2.9**	2.23	–80	**4.53**
*M. caerulea*				3.1±9.1	**16.1±2.2**	**3.2±0.4**	2.02	–83	–19.7±15.3	**17.5±5.3**	2.55	–75	**8.36**
*P. pinaster*				–4.2±13.2	**10.5±2.1**	**2.1±0.4**	2.38	–68	–29.1±16.8	**13.3±4**	2.27	–70	**1.53**
*P. aquilinum*				**90.6±39.9**	–1±12.8	–0.2±2.6	9.66	–23	59.3±54.9	4.6±11.9	9.39	–24	**0.92**
*Z. mays*	August 2015	Mature	Well-watered	7.8 ±8	0.9±0.9	0.2±0.2	1.23	–48	4.7±17.8	**2.3±1.7**	2.06	–38	**9.2**
*B. pendula*				–0.5±8.5	**9±1.7**	**1.8±0.4**	1.76	–57	–26.9±16	**11.1±3.9**	1.81	–44	<0.01
*Q. robur*				–6.5±4.6	**8.7±0.9**	**1.7±0.2**	1.06	–107	**–26.0±10.3**	**9±2.4**	1.69	–90	**16.75**
*E. gunnii*				**8.1±3.3**	**3.8±0.9**	**0.8±0.2**	0.85	–81	–10.7±8	**4±1.4**	0.95	–56	<0.01
*M. caerulea*				39.8±31.2	2±15.5	0.4±3.1	4.70	–43	14.9±51.7	5.9±16	4.58	–44	**0.77**
*P. pinaster*				**7.5±3.4**	**4.1±0.8**	**0.8±0.2**	0.80	–103	0.8±5.7	**2.5±0.7**	0.93	–91	<0.01
*P. aquilinum*				24.2±15.7	3.4±3.5	0.7±0.7	2.23	–37	10.8±25.9	3.9±4.4	2.42	–35	**1.47**
*Z. mays*	August 2015	Mature	Low-watered	**4.3±0.7**	**–1.1±0.4**	**–0.2±0.1**	0.20	–138	0.8±2.3	**0.5±0.2**	0.27	–89	<0.01
*B. pendula*				**4.7±1**	**2.2±0.4**	**0.4±0.1**	0.32	–133	–2.6±2.6	**1.6±0.4**	0.37	–82	<0.01
*Q. robur*				**5.3±2**	**2.6±0.6**	**0.5±0.1**	0.50	–172	–0.1±3.1	**1.4±0.3**	0.56	–167	**5.12**
*E. gunnii*				**8.2±2**	**4.2±0.8**	**0.8±0.2**	0.66	–109	–7.7±4.7	**3.3±0.9**	0.52	–58	<0.01
*M. caerulea*				**12.2±2**	**3.7±0.9**	**0.7±0.2**	0.53	–155	5.5±3	**2.2±0.6**	0.55	–153	**1.39**
*P. pinaster*				0.2±2.4	**4.±0.5**	**0.8±0.1**	0.70	–115	–9.4±5.1	**2.7±0.7**	1.09	–99	**15.19**
*P. aquilinum*				**2.8±1.1**	**2±0.7**	**0.4±0.2**	0.28	–101	–2.6±2.6	**1.2±0.4**	0.29	–71	<0.01

Significant (*P*<0.05) results (χ^2^) and coefficients significantly different from 0 are in bold.

**Fig. 3.**
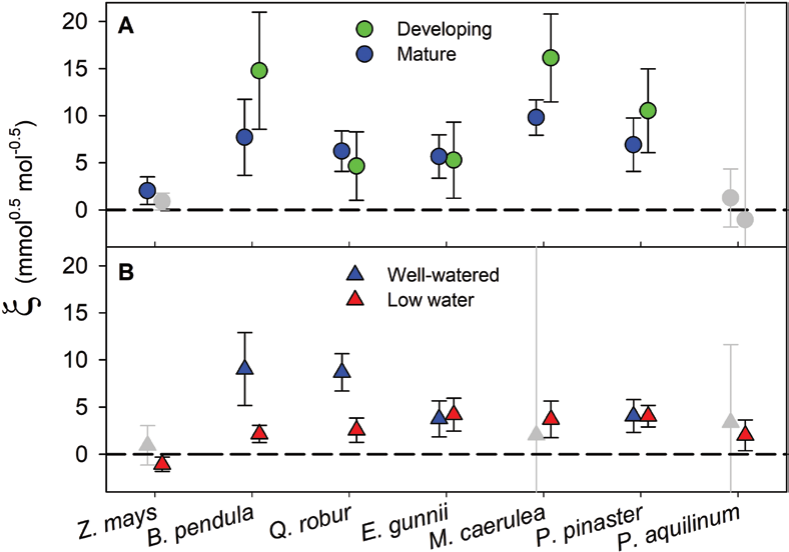
Parameter estimate (±95% CI) for the CAP formulation (ξ) fitted for (A) mature and developing leaves (June 2015 campaign, only well-watered plants) and (B) well-watered and low-watered plants (August 2015 campaign, only mature leaves), for all study species. Non-overlapping CIs indicate significant differences (*P*<0.05) among species, leaf ages, or watering treatments. Grey symbols indicate species for which the CAP fit was non-significant.

Comparison of estimated ξ (CAP formulation) values with their 95% CI showed that there were some differences among species. Deciduous angiosperm trees had the highest ξ values and the C_4_ crop (maize) the lowest (Table 2). Under well-watered conditions and in mature leaves, estimated ξ values for the four tree species were similar (Fig. 3A). We did not find clear differences in ξ values between mature and developing leaves in any species (Fig. 3A), although in *M. caerulea* ξ tended to be higher in developing than mature leaves. The low-water treatment decreased ξ in the deciduous angiosperm trees (oak and birch; Fig. 3B). For the MES formulation, analyses of homogeneity of slopes showed similar results to the comparison of the 95% CI for ξ values. Species differed significantly in their slopes ($\sqrt{E_{\max}}$) in both measurement campaigns (Table 3). Deciduous angiosperm trees had the highest slope, and the C_4_ crop the lowest, whereas all tree species had comparable slopes for mature leaves under well-watered conditions (Table 2). Leaf age did not have a significant effect on *E*_max_ and the water-stress treatment decreased *E*_max_ in angiosperm deciduous trees (Tables 2 and 3), although the relationship between *g*_sc_ and the MES index did not appear to be linear for the majority of plants under the low-water treatment (Supplementary Fig. S2).

**Table 3.** Results of the analyses of homogeneity of slopes to test for the effects of species, leaf age (mature versus developing), and watering treatment on optimization behaviour according to equation 4 (MES index is √A/1.6D(C_a_-Γ^*^))

Campaign	Effect	*F*	*P*
June	MES	205.8	**<0.001**
	MES×age	1.7	0.195
	MES×species.	3.7	**0.002**
	MES×age×species	0.6	0.742
August	MES	579.2	**<0.001**
	MES×treatment	32.2	**<0.001**
	MES×species	5.3	**<0.001**
	MES×treatment×species.	1.1	0.341

Significant effects (*P*<0.05) are indicated in bold.

**Fig. 4.**
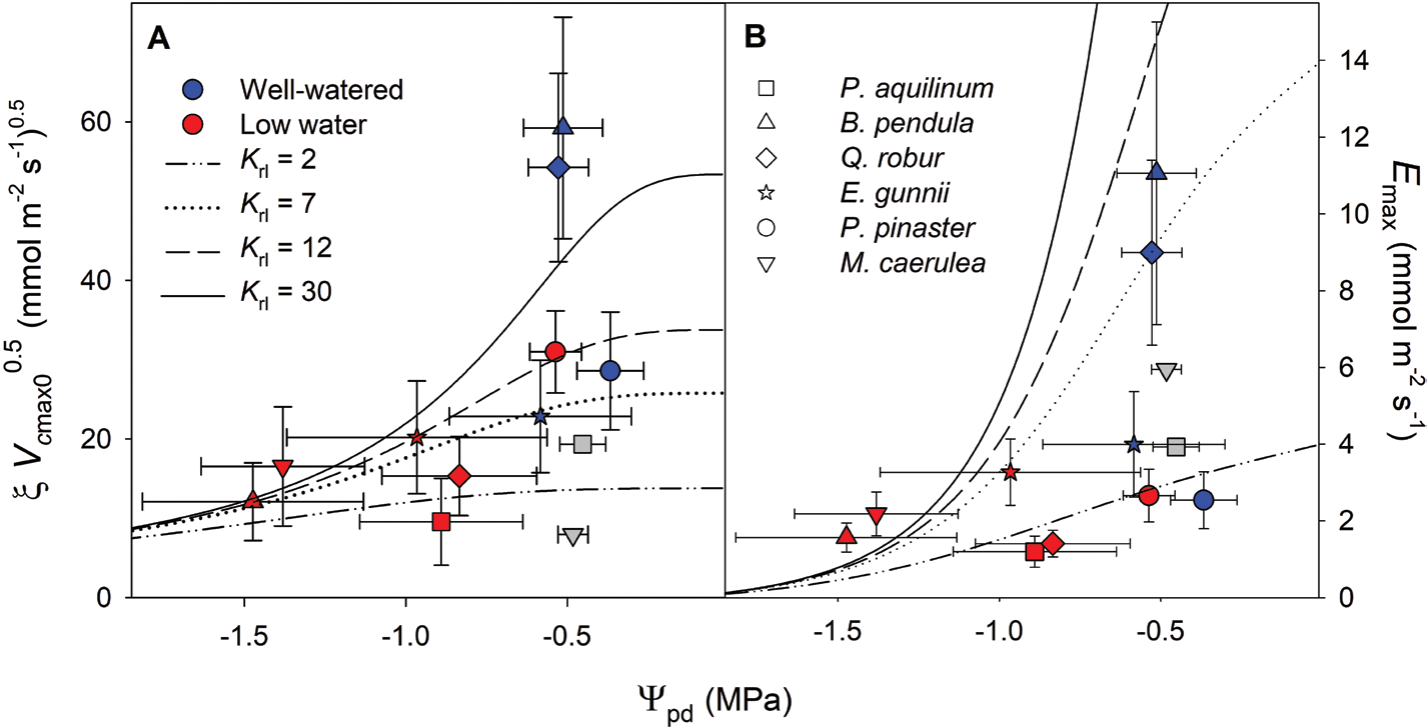
(A) Parameter estimate for the CAP model (ξ) multiplied by the square root of temperature-corrected estimated maximum carboxylation capacity (${\hat{V}}_{cmax0}$, only for the six C_3_ species) and (B) parameter estimate for the MES model (*E*_max_), both plotted against mean predawn leaf water potential (Ψ_pd_). Error bars indicate ±SE. Parameters ξ and *E*_max_ were fitted to mature leaves measured in the August campaign in well-watered and low-watered plants. Grey symbols without vertical error bars indicate species for which the CAP or MES fit were non-significant (*P*>0.05). The lines indicate the model predictions for different values of root-to-leaf xylem conductivity (*K*_rl_, mmol m^−2^ s^−1^ MPa^−1^) from equation 2 (A) and equation 4 (B).

Analyses of homogeneity of slopes for *E*_max_ and comparisons of the 95% CIs for ξ values were consistent with results of one-way ANOVAs. Under low water availability, *E*_max_ decreased (Fig. 4, *F*=8, *P*=0.015), but the species mean ξ values did not decrease significantly (Fig. 3, *F*=2.2, *P*=0.16). Leaf age did not have a significant effect on *E*_max_ (*F*=0.5, *P*=0.511) or ξ (*F*=0.4, *P*=0.561). We did not find any significant correlation between ξ and *E*_max_ with LMA or leaf thickness (*F*≤3 and *P*≥0.1 for all analyses; Supplementary Fig. S5).

When we plotted $\xi \sqrt{{\hat{V}}_{cmax0}}$ against Ψ_pd_ for well-watered and low-watered plants (Fig. 4A), we found that estimates for deciduous angiosperm trees varied greatly between watering treatments and fell close to the values predicted by the CAP formulation (equation 2) for high *K*_rl_ (50 mmol m^−2^ s^−1^ MPa^−1^). In contrast, for all other C_3_ species, $\xi \sqrt{{\hat{V}}_{cmax0}}$ did not appear to decrease notably from well-watered to low-watered plants, and our estimates fell within the CAP model predictions for lower *K*_rl_ (2–12 mmol m^−2^ s^−1^ MPa^−1^). The plot of *E*_max_ against Ψ_pd_ showed a similar pattern (Fig. 4B), but with a larger spread. Our *E*_max_ estimates for deciduous angiosperm trees fell closer to the theoretical estimates (equation 4) predicted for higher *K*_rl_.

## Discussion

In this study we compared two analytical solutions to predict *g*_sc_ based on optimization theory, assuming that the water costs of stomata opening originate from NSL to photosynthesis caused by either a reduction in carboxylation capacity (the CAP formulation) or in *g*_m_ (the MES formulation; Dewar *et al.*, 2018). We expected the CAP formulation to fit better for developing leaves and for PFTs with low-LMA leaves. Our results showed that the CAP formulation fitted better to our measurements not only for low-LMA leaves, but for all species, leaf ages, and watering regimes.

### One single formulation to predict stomatal behaviour across plant functional types

We had hypothesized that the suitability of the two formulations proposed by Dewar *et al.* (2018) would vary across species from PFTs depending on leaf morphology. Here, the variation in LMA observed among species from contrasting PFTs was consistent with previous classifications (Poorter *et al.*, 2009). Anatomical traits encompassing low *g*_m_ are usually associated with high LMA, and thus the limitation to photosynthesis imposed by *g*_m_ should increase with LMA (Flexas *et al.*, 2008; Niinemets *et al.*, 2009). We expected a better fit of the MES formulation for evergreen species (which have high LMA; Veromann-Jurgenson *et al*., 2017). However, this was not the case. Instead, the CAP formulation provided a better fit for all species, including the conifer and evergreen angiosperm trees, especially under conditions of reduced water availability (Fig. 4). This result is consistent with the midday depression observed in carboxylation capacity for all species, leaf ages, and watering levels (Supplementary Fig. S1). A better fit of the CAP over the MES formulation does not imply that *g*_m_ is not limiting photosynthesis, but rather that decreased carboxylation capacity in response to diurnal changes in Ψ_leaf_ can impose a stronger constraint on carbon gain over the timescale at which stomata operate (Franks *et al.*, 2017). This result supports our alternative expectation that in evergreen leaves, with low maximum *g*_m_ (Niinemets *et al.*, 2009; Flexas *et al.*, 2012), marginal reductions in *g*_m_ in response to daily oscillations in Ψ_leaf_, are unlikely to impose further NSL to photosynthesis over short (sub-daily) timescales. Nevertheless, the extent of our results is limited by the lack of a quantitative analysis of the relative limitations to photosynthesis (Grassi and Magnani, 2005).

Our estimates of ξ (parameter of the CAP formulation) for our study species were generally lower than but comparable to those of Lin *et al.* (2015) for their corresponding PFT. In agreement with previous classifications, we found that ξ was lowest for the C_4_ species (maize) and higher for the deciduous and herbaceous angiosperm species (Lin *et al.*, 2015; Miner *et al.*, 2017). In addition, our ξ estimates for tree saplings and potted plants were comparable to previously reported values for congeneric and closely related species grown under controlled conditions (Héroult *et al.*, 2013; Zhou *et al.*, 2013, 2014). For the crop with a C_4_ pathway (maize), we found that the CAP formulation provided a better fit (especially for mature leaves) in the June campaign, but in the August campaign it did not provide a reasonable fit. Our measurements of *A*_net_ and *g*_sc_ in this latter campaign were lower than those from the June campaign and some were nearing the limit of detection of our porometer (Fig. 1). It is possible that during this second campaign, maize plants were close to the senescent stage (Jordan-Meille and Pellerin, 2004), and a coordinated age-related decline in both *g*_sc_ and *g*_m_ could have influenced our measurements (Barbour *et al.*, 2016).

As far as we know, this is the first study to specifically test for optimal stomatal behaviour in a fern species. We found that, unless subjected to reduced water availability, stomata in the fern *P. aquilinum* did not operate according to optimization theory. The main NSL to photosynthesis in many ferns is *g*_m_ (Carriqui *et al*., 2015; Tosens *et al.*, 2016), so we expected the MES formulation to provide the best fit, but this was not the case. We suggest that in ferns, although *g*_m_ often imposes the main limitation to photosynthesis (Carriqui *et al*., 2015), at the sub-daily timescale at which stomata operate, marginal reductions in *g*_m_ with Ψ_leaf_ are unlikely to incur further costs on C gain. Additionally, although carboxylation capacity can co-limit photosynthesis in some ferns (Gago *et al.*, 2013), it is unlikely that rudimentary fern stomata are capable of exhibiting a coordinated response to minimize damage to the photosynthetic machinery (Brodribb and McAdam, 2011). Instead, collectively, our results could be interpreted as further supporting the passive hydraulic control of stomata in ferns (despite our coarse characterization of the sensitivity of *g*_sc_ to *D*_w_; Fig. 1), where changes in *D*_w_ and leaf capacitance would underlie stomatal control (Brodribb and McAdam, 2011; Martins *et al.*, 2016).

DGVM need measurable physiological traits for distinct PFTs to provide reliable estimates of vegetation–atmospheric fluxes (Miner *et al.*, 2017). Our results support that the parameter ξ (from the CAP formulation in Dewar *et al.*, 2018, and equivalent to *g*_1_ in Medlyn *et al.*, 2011) is a good candidate trait to be incorporated into DGVM to predict the coupling of carbon and water fluxes under future atmospheric conditions, in combination with other fundamental physiological traits. Parameter ξ (or *g*_1_) captures well the variability across PFTs (Fig. 3) and is commonly associated with other traits, so that it could contribute to defining common plant strategies within PFTs. For example, species with high ξ should exhibit low *V*_cmax0_ (Lin *et al.*, 2015; Hasper *et al.*, 2017; Medlyn *et al.*, 2017), as well as high |Ψ_c_| and low *K*_rl_, and hence high resistance to cavitation. Yet, we should be cautious regarding the generalization of a static ξ value for each PFT, particularly under decreasing water availability (Zhou *et al.*, 2013; Drake *et al.*, 2017). In addition, it remains to be tested how plasticity or endogenous regulation could impact ξ estimates within and across PFTs (Peguero-Pina *et al.*, 2016; de Dios *et al.*, 2017; Miner and Bauerle, 2017; Wolz *et al.*, 2017).

### Impact of water availability on optimal stomatal behaviour

We found that under low water availability, stomata behaved according to optimization theory in all our study species. The low-watering treatment had the expected physiological effects: it reduced *A*_net_, *g*_sc_, ${\hat{V}}_{cmax}$, and Ψ_pd_ relative to well-watered plants (Fig. 1, Table 1), although Ψ_pd_ did not decrease significantly in *P. pinaster* (Table 1), which maintained Ψ_pd_ well above levels required to induce stem cavitation for this species (Bouche *et al.*, 2016). Decreased *A*_net_ is directly affected by stomatal closure because of diminished substrate supply, but also by NSL (Farquhar and Sharkey, 1982). Most likely, decreased *A*_net_ under low water availability resulted from reductions in both types of NSL, that is, *g*_m_ (Théroux-Rancourt *et al.*, 2014; Wang *et al.*, 2018) and carboxylation capacity (Flexas *et al.*, 2006; Gimeno *et al.*, 2010). Here, we did not explicitly quantify the contributions of each NSL to reduced photosynthesis under water stress (Grassi and Magnani, 2005; Zhou *et al.*, 2013; Drake *et al.*, 2017) and we did not measure *g*_m_. Yet, we found that ${\hat{V}}_{cmax0}$ decreased at midday and the CAP formulation provided the best fit under both watering levels (Fig. 2). Thus, we suggest that reduced carboxylation capacity is likely to be a major driver underlying rapid (sub-daily) stomatal regulation, irrespective of water availability.

We observed some differences in stomatal behaviour among species from the six PFTs as a consequence of reduced water availability. The marginal water cost to C gain (ξ or *g*_1_) was conserved under low water availability, except for the two deciduous angiosperm trees, for which ξ decreased under low water availability. Conserved ξ or *g*_1_ under decreasing water availability has previously been reported for species with a large drought-tolerance range, while more drought-sensitive species were capable of modulating their marginal water cost under water stress (Héroult *et al.*, 2013; Zhou *et al.*, 2013; Gimeno *et al.*, 2016; Miner and Bauerle, 2017). For the fern, we found a tight coupling of *g*_sc_ and *A*_net_ according to optimization theory only under low water availability, suggesting that the hydro-passive mechanism underlying the stomatal response to increasing *D*_w_ might be functional only below a certain water availability threshold (Martins *et al.*, 2016). According to the CAP formulation, Dewar *et al.* (2018) predict a larger decrease in $\xi \sqrt{{\hat{V}}_{cmax0}}$ with Ψ_leaf_ for species with a higher root-to-leaf conductivity (*K*_rl_). Our results support this prediction (Fig. 4), as we found that for the two deciduous angiosperm trees, which were expected to exhibit high *K*_rl_ (Domec *et al.*, 2017), our estimates of $\xi \sqrt{{\hat{V}}_{cmax0}}$ fell close to the CAP model predictions for the higher range of *K*_rl_. For the conifer (*P. pinaster*), the water-stress treatment did not reduce Ψ_pd_ and our estimates of $\xi \sqrt{{\hat{V}}_{cmax0}}$ from low-watered and well-watered plants overlapped (Fig. 4), a response that typifies isohydric behaviour, consistent with previous observations for this species (Ripullone *et al.*, 2007).

### Optimal stomatal behaviour in developing leaves

We found that optimal stomatal behaviour in developing leaves mimicked that of their mature counterparts (Fig. 3). Contrary to our expectations, we did not find lower *A*_net_ in developing leaves. Similar *A*_net_ would imply that in developing leaves respiratory costs associated with construction would be compensated for in mature leaves with greater maintenance costs (Zhou *et al.*, 2015). Alternatively, or in addition, developing leaves would compensate for extra respiratory costs with greater carboxylation capacity (Medlyn *et al.*, 2002; Rajaona *et al.*, 2013; Locke and Ort, 2014), but this latter explanation is not supported by our estimates of carboxylation capacity using the one-point method (Supplementary Fig. S1) (De Kauwe *et al.*, 2016). More importantly, the CAP formulation predicted similar coupling between *g*_sc_ and *A*_net_ with no clear differences in the marginal water cost to C gain (ξ or *g*_1_) between mature and developing leaves. The CAP formulation fitted best for both leaf ages, except for the developing leaves of pine and oak, although the CAP formulation also successfully predicted *g*_sc_ in the developing leaves of these species. This result supports our initial expectation that the photosynthetic apparatus of developing leaves would be sensitive to diurnal oscillations in Ψ_leaf_ and their stomata would operate to minimize the likelihood of dehydration damage (Chaves *et al.*, 2002). Similar stomatal behaviour for both leaf ages suggests that developing leaves might not be less tolerant to dehydration, although the underlying protective mechanisms could vary. For example, it has been shown that developing leaves can tolerate similar water stress to mature leaves, albeit at a greater carbon cost as they rely on the accumulation of compatible solutes (Sperdouli and Moustakas, 2014). This strategy would compensate for a lack of morphological adaptation expressed later in ontogeny. Furthermore, our results could also suggest that *g*_m_ limits photosynthesis in mature and developing leaves to a similar extent and that *g*_m_ does not necessarily decrease during leaf development. Developmental changes that result in lower *g*_m_, such as thicker cell walls, could be partially compensated, for example, with an increase in the chloroplast per surface area ratio (Evans *et al.*, 2009). Overall, our results did not support that the type of formulation and the marginal water cost to C gain in the developing stage would differ from those applied to mature canopies. Instead, our results indicated that stomatal behaviour was conserved along leaf ontogeny and across PFTs, despite potentially large differences in construction costs (Wright *et al.*, 2004; Onoda *et al.*, 2017). So far, DGVM have ignored possible changes in stomatal behaviour with leaf ontogeny, even for deciduous forests in temperate regions (Keenan *et al.*, 2013). Our study, together with other recent observations (Macinnis-Ng *et al.*, 2017), suggest that the CAP formulation could serve as a homogeneous approach to predict *g*_sc_ under changing environmental conditions, irrespective of leaf ontogenetic stage.

## Conclusions

We have shown that the optimal formulation assuming that water costs to carbon gain arise from reduced carboxylation capacity (CAP) provides a suitable fit for predicting stomatal behaviour irrespective of leaf ontogenetic stage, PFT, and water availability. Our results do not imply that NSL to photosynthesis are restricted to reduced carboxylation capacity. Instead, we argue that sub-daily marginal changes in *g*_m_ are unlikely to be influencing the rapid stomatal responses to atmospheric drought. Our results suggest that the CAP formulation successfully captures the co-variation of *g*_sc_ and *A*_net_ with increasing *D*_w_, irrespective of PFT, water stress, or leaf ontogenetic stage, for the timescale at which stomata operate. Our data also support that the use of a single-parameter CAP formulation is valid for most PFTs, with the caveat that the CAP parameter (ξ or *g*_1_) should not be assumed to be static in response to water availability for all PFTs, particularly for those most sensitive to drought.

## Supplementary data

Supplementary data are available at *JXB* online.

Protocol S1. Estimated carboxylation capacity with the one-point method.

Fig. S1. Estimated carboxylation capacity.

Fig. S2. Internal to ambient CO_2_ molar ratio against vapour pressure deficit.

Fig. S3. Stomatal conductance plotted against the indexes of the two formulations.

Fig. S4. Leaf mass per area and leaf thickness for all species and two leaf ages.

Fig. S5. Model slope parameters plotted against leaf mass per area and leaf thickness.

Supplementary Protocol S1 and Figures S1-S5

## Abbreviations:

*A*_net_: net photosynthesis
*C*_a_: ambient CO_2_ concentration
*C*_i_: leaf intercellular CO_2_ concentration
DGVM: dynamic global vegetation models
*D*_w_: vapour pressure deficit
E: transpiration
*g*_m_: mesophyll conductance
*g*_s_: stomatal conductance to water
*g*_sc_: stomatal conductance to CO_2_
*K*_rl_: root-to-leaf conductivity
*K*_sl_: soil-to-leaf conductivity
LMA: leaf mass per area
NSL: non-stomatal limitations
PFT: plant functional type
Γ*: CO_2_ photorespiratory compensation point
Ψ_leaf_: leaf water potential
Ψ_pd_: predawn leaf water potential
