# Supplementary material for: A novel optimization approach incorporating non-stomatal limitations predicts stomatal behaviour in species from six plant functional types
Source: J Exp Bot. 2019 Feb 4;70(5):1639–51. doi: 10.1093/jxb/erz020 (PMC6411372; doi:10.1093/jxb/erz020)
Supplement: Supplementary Protocol S1 and Figures S1-S5 [file erz020_suppl_supplementary_protocol_s1_figures_s1-s5.pdf]

**SUPPLEMENTARY DATA**

**Running title:** Stomatal optimisation behaviour across plant functional types

# A novel optimization approach incorporating non-stomatal limitations predicted stomatal behaviour on species from six plant functional types

Teresa E. Gimeno[1,2,3*], Noelia Saavedra[1,4], Jérôme Ogée[1], Belinda E. Medlyn[5] & Lisa Wingate[1]

[1]INRA, UMR ISPA, 33140, Villenave d'Ornon, France

*Present address*: [2]Basque Centre for Climate Change (BC3), 48940, Leioa, Spain

[3]IKERBASQUE, Basque Foundation for Science, 48008 Bilbao, Spain

[4]*Present address*: Department of Forest Ecology and Management, Swedish University of Agricultural Sciences (SLU), Skogsmarksgränd 17, 907 36, Umeå, Sweden

[5]Hawkesbury Institute for the Environment, Western Sydney University, Locked bag 1797, Penrith NSW 2751, Australia

[*] Corresponding author:

Teresa E. Gimeno

E-mail: teresa.gimeno@bc3research.org

Phone: +34 94 401 46 90

Co-authors email addresses: NS (noelia.saavedra@slu.se), JO (jerome.ogee@inra.fr), BEM (b.medlyn@westernsydney.edu.au), LW (lisa.wingate@inra.fr)

**Supplementary methods:** Estimated carboxylation capacity with the one-point method.

**Figure S1.** Estimated carboxylation capacity.

**Figure S2.** Internal to ambient $CO_2$ molar ratio against vapour pressure deficit.

**Figure S3.** Stomatal conductance plotted against the indexes of the two formulations.

**Figure S4.** Leaf mass per area and leaf thickness for all species and two leaf ages.

**Figure S5.** Model slope parameters plotted against leaf mass per area and leaf thickness.

## SUPPLEMENTARY DATA

**Supplementary methods:** Estimated carboxylation capacity under contrasting water availabilities and leaf ages

Carboxylation capacity for the six $C_3$ species was estimated with the 'one-point' method (De Kauwe *et al.*, 2016), based on the original biochemical model for $C_3$ photosynthesis (Farquhar *et al.* 1980). We used measurements of gas-exchange at saturating light intensity and ambient $CO_2$ concentration to estimate carboxylation capacity ($\hat{V}_{\mathrm{cmax}}$) according to:

Eq. A1

$$\hat{V}_{\mathrm{cmax}} = \frac{A_{\mathrm{net}}}{\frac{C_{\mathrm{i}} - \Gamma^*}{C_{\mathrm{i}} + K_{\mathrm{m}}} - 0.015}$$

where $A_{net}$ (µmol m$^{-2}$ s$^{-1}$) is net photosynthesis, $C_i$ (µmol mol$^{-1}$) is the intercellular $CO_2$ concentration, $\Gamma^*$ (µmol mol$^{-1}$) is the $CO_2$ photo-respiratory compensation point (according to Bernacchi *et al.*, 2001) and $K_m$ is the effective Michaelis-Menten constant for the carboxylation reaction by Rubisco taking into account the competitive inhibition effect by $O_2$. We estimated temperature-corrected $\hat{V}_{\mathrm{cmax}}$ from our mid-morning (maximum) and midday (minimum) measurements of $A_{net}$ and $C_i$, for the six $C_3$ species, both leaf ages and water availabilities.

As expected, $\hat{V}_{\mathrm{cmax}}$ was lower when estimated from measurements at midday than mid-morning ($F > 25$, $p < 0.001$, for both campaigns) and species differed in $\hat{V}_{\mathrm{cmax}}$ ($F > 3$, $P < 0.05$, for both campaigns). We found that mature leaves had higher $\hat{V}_{\mathrm{cmax}}$ than developing ones ($F = 10.9$, $p = 0.001$, Figure S1a), but this difference was species specific ($F = 2.2$, $p = 0.065$). Also as expected, overall $\hat{V}_{\mathrm{cmax}}$ was marginally higher in well- than low-watered plants ($F = 2.9$, $p = 0.097$, Figure S1b).

**Bernacchi CJ, Singsaas EL, Pimentel C, Portis AR, Long SP**. 2001. Improved temperature response functions for models of Rubisco-limited photosynthesis. *Plant, Cell and Environment* **24**, 253-259.

**De Kauwe MG, Lin YS, Wright IJ *et al.*** 2016. A test of the 'one-point method' for estimating maximum carboxylation capacity from field-measured, light-saturated photosynthesis. *New Phytologist* **210**, 1130-1144.

**Farquhar GD, Caemmerer SV, Berry JA**. 1980. A Biochemical-Model of Photosynthetic $CO_2$ Assimilation in Leaves of $C_3$ Species. *Planta* **149**, 78-90.

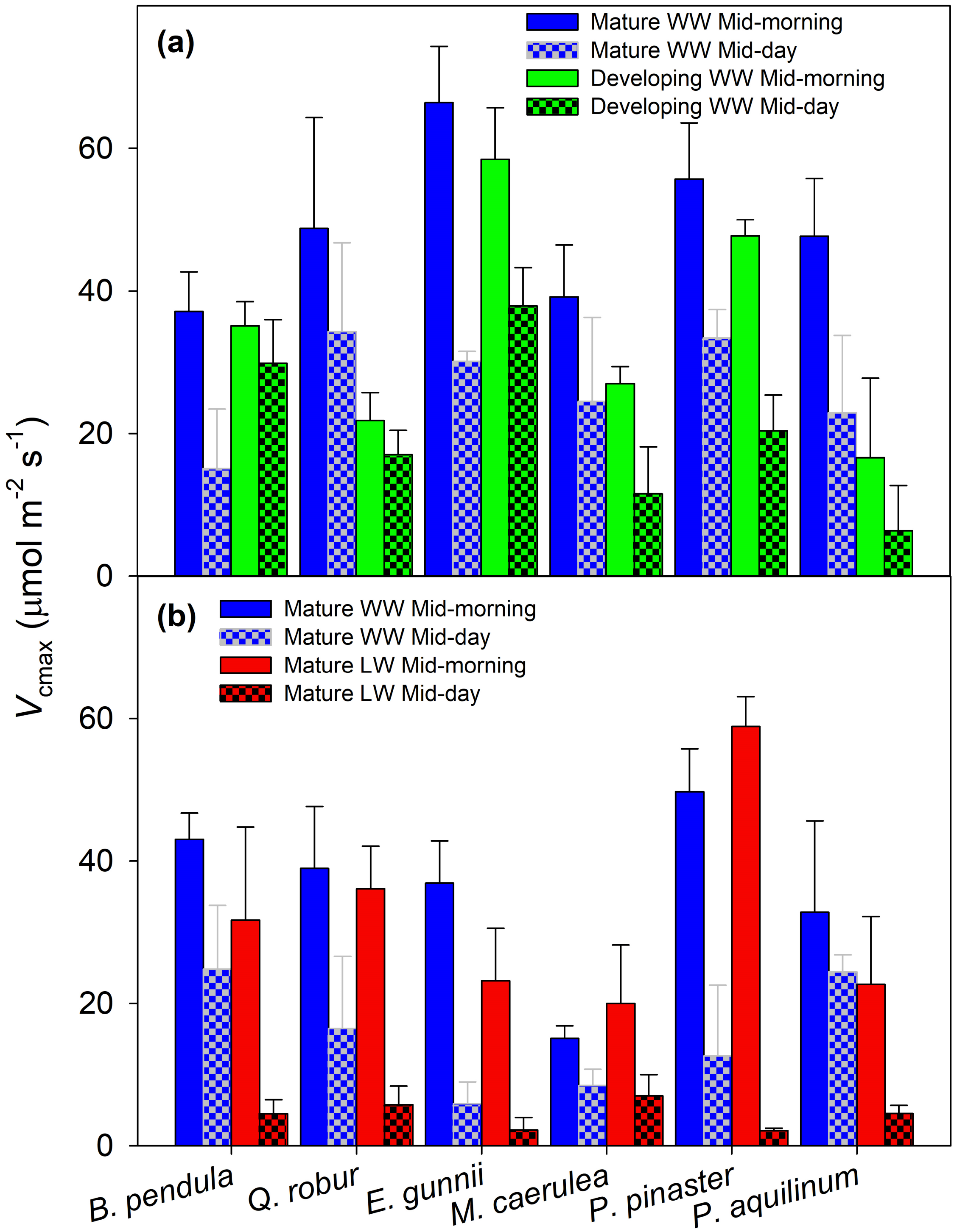


**Figure S1.** Mid-morning (maximum) and midday (minimum) estimated carboxylation capacity ($\widehat{V}_{\text{cmax}}$ ± se) with the one-point method (temperature-corrected) fitted for **(a)** mature and developing leaves (June-2015 campaign) and **(b)** for well-watered (WW) and low-watered (LW) plants (August-2015 campaign), for the six $C_3$ study species.

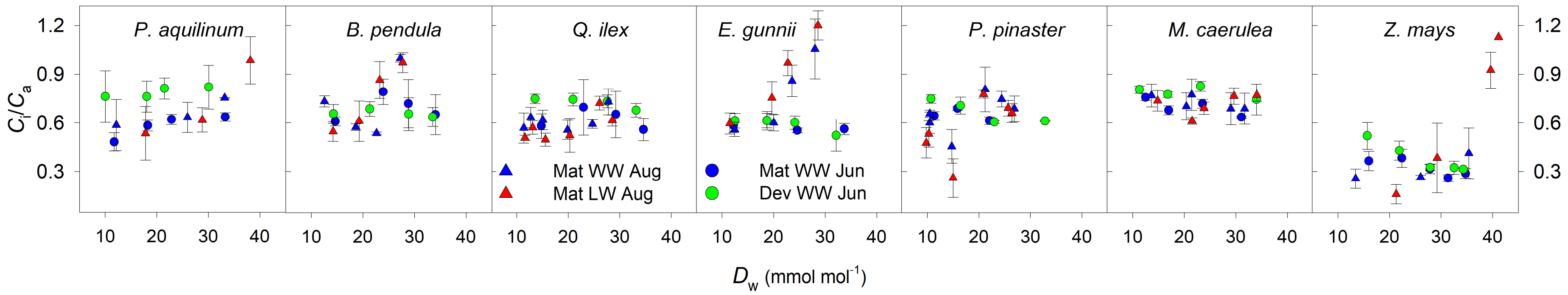


**Figure S2.** Mean (±se, $n$ = 3-5) ratio of intercellular to ambient $CO_2$ concentration ($C_i/C_a$) along a vapour pressure deficit ($D_w$) in mature (Mat.) and developing (Dev.) leaves and in well-watered (WW) and low watered (LW) plants. Circles and triangles depict measurements from the June-2015 and August-2015 campaigns, respectively. Different colours correspond to measurements on WW (blue and green) vs. LW (red) plants or to measurements on mature (blue and red) or developing (green) leaves.

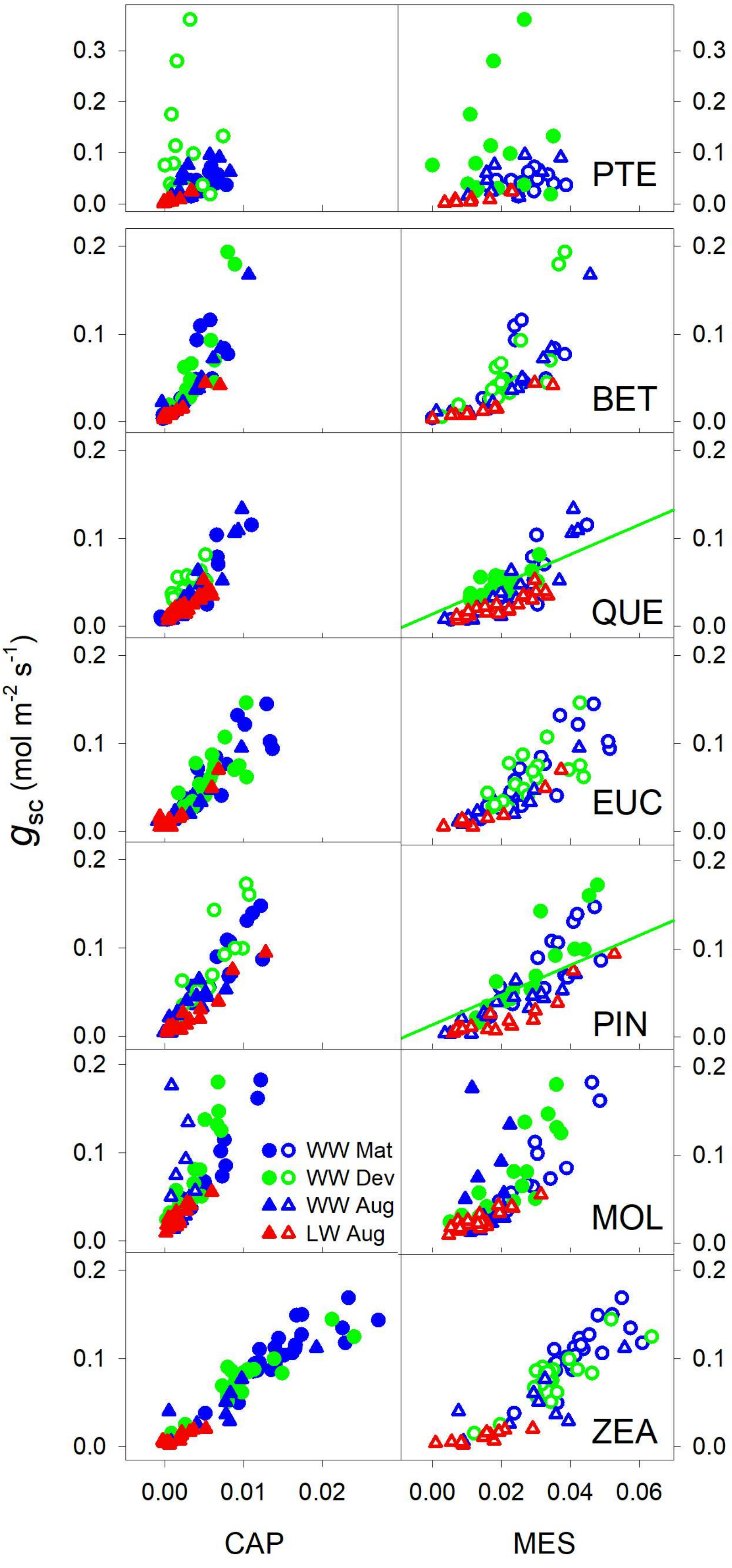
PTE
BET
QUE
EUC
PIN
MOL
ZEA
WW Mat
WW Dev
WW Aug
LW Aug
$g_{sc}$ (mol m$^{-2}$ s$^{-1}$)
CAP
MES
0.00
0.01
0.02
0.00
0.02
0.04
0.06
0.0
0.1
0.2
0.3

**Figure S3.** Relationship between stomatal conductance to $CO_2$ ($g_{sc}$) and the CAP index [$A_{net}/\sqrt{D_w}(C_a-\Gamma^*)$, in mol$^{1.5}$ mmol$^{-0.5}$ m$^{-2}$ s$^{-1}$, Eq. 1] or the MES index [$\sqrt{(A_{net}/1.6D_w(C_a-\Gamma^*)}$, in mol mmol$^{-0.5}$ m$^{-1}$ s$^{-0.5}$, Eq. 4]. Circles and triangles depict measurements from the June-2015 and August-2015 campaigns, respectively. Different colours correspond to measurements on well-watered (WW, blue and green) vs. low-watered (LW) plants (red) or to measurements on mature (blue and red) or developing (green) leaves. Filled symbols denote the fit with the lower AIC. For those species and treatment combinations where the linear regression ($g_{sc}$ vs. MES index) was significant ($p$ < 0.05) and $AIC_{MES} < AIC_{CAP}$ (filled symbols on the right panel) lines represent the relationship between $g_{sc}$ and the MES index. Note the change in scale for $g_{sc}$ in the uppermost panels (PTE). Species codes are: *P. aquilinum* (PTE), *B. pendula* (BET), *Q. robur* (QUE), *E. gunnii* (EUC), *P. pinaster* (PIN), *M. caerulea* (MOL) and *Z. mays* (ZEA).

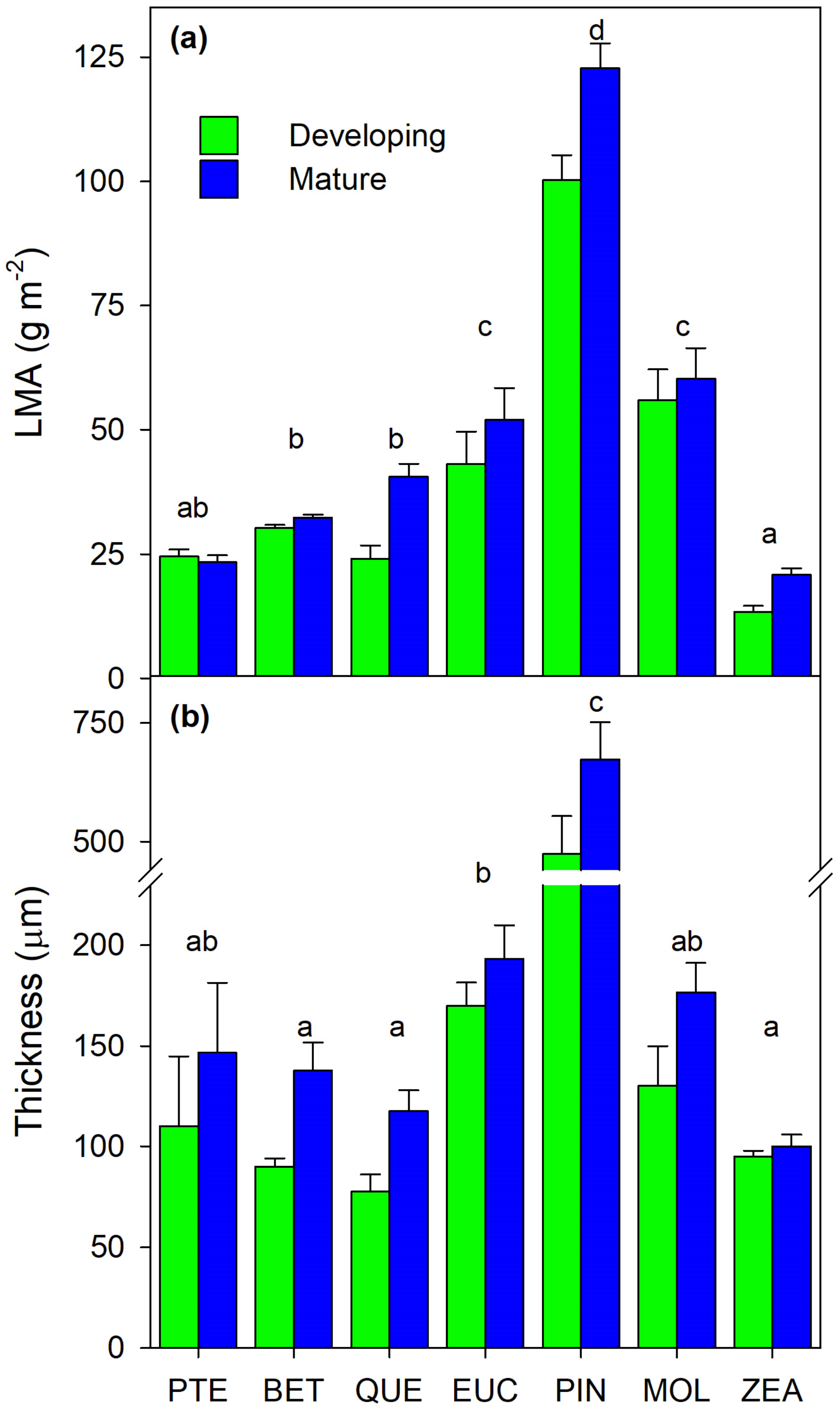


**Figure S4**. Mean (±se, *n* = 5) **(a)** leaf mass per area (LMA) and **(b)** leaf thickness for the study species. Species codes are: *P. aquilinum* (PTE), *B. pendula* (BET), *Q. robur* (QUE), *E. gunnii* (EUC), *P. pinaster* (PIN), *M. caerulea* (MOL) and *Z. mays* (ZEA). Letters indicate significant differences among species. Developing leaves had significantly lower values than mature leaves for both traits.

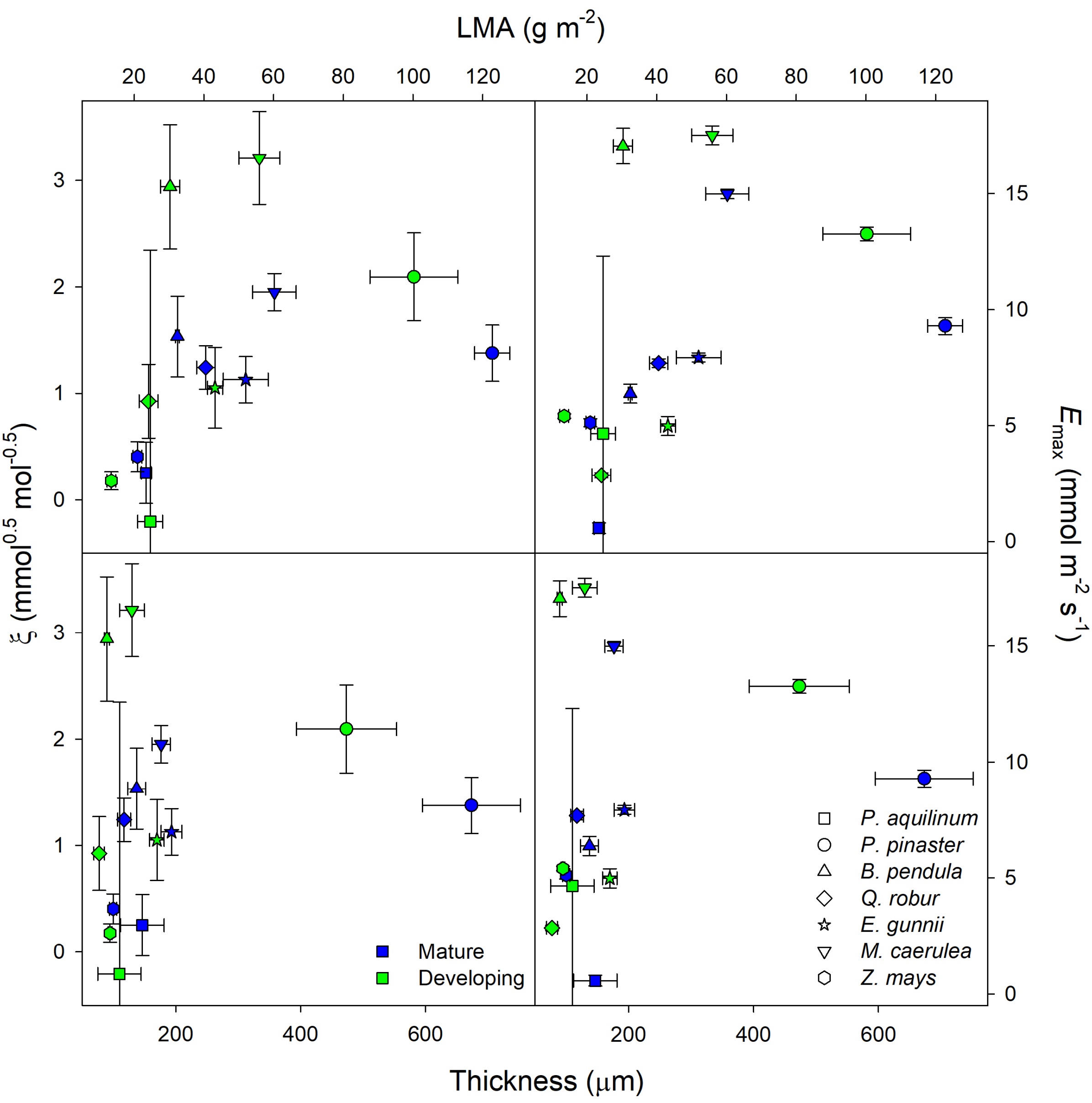


**Figure S5.** Estimated parameters (±se) for the two formulations of the optimisation model assuming that the cost of stomatal opening arises either from reduced carboxylation capacity (CAP, ξ) or reduced mesophyll conductance (MES, $E_{max}$) plotted against mean (±se) leaf mas per area (LMA) or leaf thickness, for the seven study species in mature and developing leaves (June-2015 campaign).
